# An Integrative Approach to the Current Treatment of HIV-Associated Neurocognitive Disorders and the Implementation of Leukemia Inhibitor Factor as a Mediator of Neurocognitive Preservation

**DOI:** 10.3390/life13112194

**Published:** 2023-11-11

**Authors:** Andrés De Freitas-Suarez, Natalia Espinosa-Ponce, Natalia Alvarez-Roger, Arianna Iris Cabrera-Suarez, Guillermo Jiménez-Jordán, Rocio Vega-Roman, Mikhail Inyushin, Janaina M. Alves

**Affiliations:** 1 Department of Pharmacy, University of Illinois—Chicago, Chicago, IL 60608, USA; adefre2@uic.edu; 2 Department of Microbiology and Immunology, Universidad Central del Caribe School of Medicine, Bayamón, PR 00960, USA; natalia.espinosa@upr.edu (N.E.-P.); arianna.cabrera@upr.edu (A.I.C.-S.); 3 Department of Medicine, Universidad Central del Caribe, Bayamón, PR 00956, USA; natalia.alvarez9@outlook.com (N.A.-R.); 120rvega@uccaribe.edu (R.V.-R.); 4 Department of Medicine, Ponce Health Sciences University, Ponce, PR 00732, USA; guillermo.jimenez@upr.edu; 5 Department of Physiology, Universidad Central del Caribe School of Medicine, Bayamón, PR 00960, USA; mikhail.inyushin@uccaribe.edu

**Keywords:** HIV, HAND, neurocognitive disorders, LIF, neuroprotection

## Abstract

HIV-associated neurocognitive disorders (HANDs) continue to impact patients despite antiretroviral therapy. A combination of antiretroviral therapies can diminish the HIV viral load to near undetectable levels, but fails to preserve neurocognitive integrity. The cytokine leukemia inhibitory factor (LIF) has shown neuroprotective properties that could mitigate neurodegeneration in HANDs. The LIF promotes neurogenesis, neural cell differentiation, and survival. Combination antiretroviral therapy reduces severe forms of HANDs, but neurocognitive impairment persists; additionally, some antiretrovirals have additional adverse neurotoxic effects. The LIF counteracts neurotoxic viral proteins and limits neural cell damage in models of neuroinflammation. Adding the LIF as an adjuvant therapy to enhance neuroprotection merits further research for managing HANDs. The successful implementation of the LIF to current therapies would contribute to achieving a better quality of life for the affected population.

## 1. Introduction

### 1.1. Defining the Nature of LIF

The leukemia inhibitory factor (LIF) is a fascinating complex cytokine within the human body. It is present in a grand variety of biological processes, and it demonstrates a broad array of roles. These processes include maintaining the pluripotency of stem cells, tissue development, inflammation, and blood vessel formation [1]. Over the last decade, researchers have been drawn to LIF due to its diverse range of applications. The main area of interest is the role it plays in the central nervous system. Many researchers see the LIF as a potential component for therapeutic remedies for neurodegenerative diseases such as HIV-associated neurocognitive disorders (HANDs) and Alzheimer’s disease.

The LIF is classified as a member of the IL-6 family of cytokines. All of the cytokines that make up the IL-6 family use gp130 as a receptor subunit. These cytokines are involved in very different roles and processes at a microscopic level throughout the human body. It has been proven that LIF plays a role in maintaining the pluripotency of embryonic stem cells (ESCs) along with a fellow member of the IL-6 family, Oncostatin M (OSM). It has been shown that ESCs maintain their pluripotency when either the LIF or OSM is bound to the gp130 receptor subunit, essentially demonstrating that the LIF plays a massive role in determining whether ESCs differentiate into more specialized cells or they retain their pluripotency for a longer period of time [2]. This confirms that the LIF is responsible for essential mechanisms during the earliest development of a human embryo. Studies have also shown that the LIF can promote the differentiation of adult exocrine pancreatic cells into insulin-producing beta cells in vitro [3], suggesting that the LIF plays an active role in the development of pancreatic tissue in the human body and in the generation of functional pancreatic cells. It is important to highlight these functions of the LIF cytokine in order to understand its involvement in biological processes that span various systems of the human body.

The LIF has also been demonstrated to be a part of both proinflammatory responses and anti-inflammatory responses in physiological processes. It has been implicated as a proinflammatory agent in conditions such as allergic rhinitis, ulcerative colitis, and pancreatic carcinoma [4,5,6]. On the other hand, the LIF has also been shown to be associated with anti-inflammatory responses in the human body. It has been shown to repress the GnRH gene, which codes for the proinflammatory cytokine GnRH [7]. The duality of LIF in either pro- or anti-inflammatory responses is important to understand because it further highlights that the LIF is just a single part of many more complex mechanisms occurring within the inflammation. The precise role of the LIF in inflammation depends on many external factors and requires more investigation in order to be fully understood.

Another very crucial feature of the LIF is its role in angiogenesis. Angiogenesis is defined as the growth of new blood vessels [8]. One study demonstrates that the LIF promotes the regeneration of the myocardium and ensures the survival of cardiomyocytes after a heart attack. This results in improved angiogenesis and a larger presence of bone-marrow-derived cells in the heart [9]. The LIF has also been associated with the formation of blood vessels in the endometrium. The increase in the LIF in the endometrium is related to improved endometrial receptivity, the early formation of blood vessels, and facilitated embryo adhesion [10]. Understanding the LIF’s role in angiogenesis in structures such as the heart and endometrium is necessary when it comes to comparing this to its role in the CNS. It is significant because it could imply that the LIF plays a role in the regulation of the blood vessels that supply the CNS and in structures such as the blood–brain barrier (BBB).

The LIF has also been shown to be significantly involved in many mechanisms within the peripheral nervous system (PNS). The LIF has been observed to promote cell differentiation, growth, and survival in motor neurons and has been studied in both in vitro and in vivo models [11]. The previous studies further confirm that the LIF is highly involved in neuroprotection in different parts of the nervous system. It has been proven that the LIF takes part in mechanisms that promote the growth and regeneration of sensory neurons around the PNS after nerve damage [12]. A study performed with mice confirmed that the LIF promotes the reparative regeneration of peripheral nerves [13]. The study proved to be impactful due to it demonstrating that the LIF can act as both a polyfunctional cytokine and a neurotrophic factor. All these data can validate that the LIF is an active member of many pathways and mechanisms that form part of the PNS.

The scope of this review is aimed towards elucidating the role the LIF plays in the CNS. It is an integral component of the IL-6 family for processes such as neuronal development, survival, and protection [14,15]. It has been demonstrated that the LIF is present in significant amounts within the cerebrospinal fluid (CSF) and brain parenchyma [16]. This suggests that it is vital for different processes that take place in the CNS. Interestingly, there are large quantities of the LIF stored and released by astrocytes in the CNS. The astrocytes increase the expression of the LIF whenever there is an event that causes neuronal damage. Although the regulatory mechanism of how the astrocytic LIF is expressed is still not clear, it confirms that the LIF is present whenever there is an event of neuronal damage [17]. This would heavily suggest that the LIF has some kind of neuroprotective capabilities. It has also been demonstrated that the LIF plays a critical role in appropriate glial responses, remyelination, and the preservation of neural conductance after mild traumatic injury to the brain [15]. This is another piece of evidence that would lend credence to the idea that the LIF exhibits neuroprotective qualities within the central nervous system. Another important aspect of this study is that it confirms that the LIF is an important element of both neurons and glial cells within the CNS. All these data confirm that the LIF is an important component of both the CNS and PNS. This is very exciting because this opens up even more doors when it comes to assessing the LIF’s candidacy as a therapeutic agent to treat different neurocognitive diseases.

The LIF also plays a critical role in the regulation of neurogenesis. It has been shown that the LIF regulates the formation of neurons in various regions of the brain, including the olfactory bulb and subventricular zone. The LIF has been found to discourage neurogenesis in these studies by acting directly on neural stem cells (NSCs) and causing them to stay in their undifferentiated state for a longer period of time [18,19]. The results suggest that the presence or absence of the LIF is a crucial factor when it comes to determining the levels of neurogenesis in the nervous system. These findings are important because they suggest that the LIF in combination with other factors could be a therapeutic option to promote regeneration in brain tissue. The LIF, along with other factors such as the ciliary neurotrophic factor and other IL-6 cytokines, plays a very important role in the regulation of neurogenesis in the CNS. All of these factors work together to increase or decrease the degree of neurogenesis depending on the needs of the CNS [20,21]. The LIF has been found to be secreted by both astrocytes and pericytes along the CNS. These two types of cells are very involved in both neurogenesis and vascular functions of the CNS [22,23]. This information serves as further confirmation of the notion that the LIF plays a critical role in many processes related to neurogenesis within the brain.

Another very important contribution that the LIF offers to the CNS is its role in cell differentiation. The LIF has been classified as a neuropoietic cytokine that regulates the differentiation of different cell types within the brain [24]. A neuropoietic cytokine is defined as one that plays a role in the control of neuronal, glial, or immune responses to an injury or disease [25]. The LIF has also been shown to promote the differentiation, survival, and proliferation of neural progenitor cells (NPCs) in vitro [26,27]. This evidence would suggest that the LIF is very involved in the early stages of the development of the CNS. The LIF has also been implicated in astrocyte differentiation within the CNS [28]. This is significant because it reveals that the LIF is involved in the differentiation of both neurons and glial cells. It is evident that the LIF is present in many different aspects and processes of all kinds throughout the brain. This is relevant because it contributes to the possibility of it being developed as a therapeutic agent for different types of neurodegenerative diseases such as HIV and Alzheimer’s syndrome.

### 1.2. LIF Pathway

As mentioned beforehand, the LIF is a cytokine known to have a wide range of functions in different organs such as the bones, liver, kidneys, and CNS, particularly in embryonic cells. The signaling pathways induced by this cytokine are similar among many cells, JAK/STAT 3, PI3K/AKT, and MAPK, which contribute to self-renewal, survival, and differentiation, respectively. The LIF receptor (LIFR) consists of two signaling chains, gp130 and LIFRb [29], which are intracellular domains that are associated with JAK1, a tyrosine kinase known to initiate the signaling cascade. When the extracellular LIF induces the receptor, JAK1 is subsequently transphosphorylated and activated [29]. This results in the phosphorylation of five tyrosine on each chain of the receptor, which recruits more signaling proteins like STAT 3 [30]. STAT 3 is a transcription factor proven to be the mediator of most cellular effects as it translocates to the nucleus and upregulates the transcription of the cytokine important for the self-renewal of cells [30]. One target of STAT 3 is the protein SOCS3, which negatively modulates the JAK/STAT3 and MAPK pathways. On the other hand, the pathway that induces cell survival is PI3/AKT. After the stimulation of the LIF, JAK 1 was associated with subunit p85 of phosphatidylinositol (3,4,5)-triphosphate from phosphatidylinositol (4,5)-diphosphate (PI3 kinase) [29]. This reaction leads to the activation of serine/threonine kinase (AKT), initiating other signaling cascades that ultimately allow cell survival. The MAPK pathway, usually required for differentiation, starts when the SHP2 protein is phosphorylated by JAK1, stimulating Ras/Raf signaling cascade, resulting in the activation of MAPK, a transcription factor known to promote stem cells to become organ-specific. Modulating these three pathways, the LIF stimulates a mixture of effects depending on the cell’s fate.

The effects of the LIF have been widely studied in the central nervous system because it exerts many important functions via the activation of LIFR/gp130, particularly on the development of neural stem cells and neuroglia. The LIF has been proven to stimulate the production, maturation, and survival of oligodendrocytes as well as induce the differentiation of the astrocyte progenitor cell line into GFAP+ astrocytes [29]. LIF receptor signaling provides different options for CNS development, enhancing or inhibiting the neural cells depending on the cell environment and tissue demands. Previous studies on animal models of neurodegeneration have shown that the LIF promotes the regeneration of damaged tissue as neurons increase the expression of this cytokine in response to injury [31]. Although the LIF can have different kinds of expression in our body, most notably the CNS, its role in neurogenesis has been widely studied in structures such as the primary visual cortex, olfactory receptor neurons, and the hippocampus. The LIF has been proven to have a fundamental role in enhancing the proliferation and myelinization of mature and progenitor cells of astroglia in the hippocampus, decreasing the degradation of neurons in models of inflammation and neurodegeneration [32]. This has led to the study of the LIF as a protective factor against neurocognitive disorders.

### 1.3. JAK-STAT Everlasting Relation with HIV-1

The JAK-STAT signaling pathway mediates the important role of the innate immune response against a wide range of viral infections, including HIV-1 [30]. The mechanisms of antiviral immune signaling are a complex, orchestrated process that leads to the activation of many proinflammatory cytokines like interferons (INFs), macrophages, and many other immune cells to inhibit viral replication and stimulate the humoral immune response to prevent the propagation of the infection [32]. Viruses have evolved through the years and have many strategies to evade our immune system, but previous studies have shown that the JAK-STAT pathway is one of the most relevant in the regulation of local and systemic inflammation in response to viral infections. Once the viral particles are recognized by the antigen-presenting cells, interferons are released and stimulate the phosphorylation of STAT, which starts the cytokine cascade. Many viruses antagonize the functions of STAT, allowing chronic infections to develop. One of the many viruses that cause persistent infection is HIV-1, which has been characterized to be a modulator of the JAK-STAT pathway via Vpu and Nef proteins [30], blocking STAT phosphorylation following INF-alpha stimulation, to enable its replication without the antiviral action of STAT. Other accessory proteins are Vif, Vpu, and Vpr, which play a role in the recruitment of proteins for ubiquitination and proteasomal degradation [30]. Vif has been proven to interfere with IFN-alpha signaling via the degradation of STAT 1 and STAT 3, inhibiting the inflammatory cascade and facilitating virus survival [32].

The upregulation of JAK-STAT signaling by viral infections shares similar characteristics to that used by the LIF to induce neuroprotection once cells become infected by HIV-1, for example. The LIF counteracts the neurotoxic effect of such inhibiting cytokines like Nef and Vip by modifying the STAT 3 pathway for neuronal renewal and survival instead of the detrimental effects of those proinflammatory cytokines [29]. This explains the particular interest in studying the effects of the LIF as a neuroprotective factor against the long-term sequelae of HIV manifestation in the CNS.

### 1.4. Neuroprotective Properties of LIF

The neuroprotective role of the LIF has been more widely studied in recent years as a possible therapeutic agent against many diseases that have detrimental consequences on our central nervous systems, like HIV-1 infection and its associated neurocognitive disorder. Many studies have recognized the LIF as a key player in modulating the neuroimmune balance in our CNS in two main ways: stimulating neuronal renewal and survival and modulating inflammation [33]. The brain has developed mechanisms for self-protection, and upon infection or brain injury, endothelial cells have been shown to release the LIF to promote the differentiation of neuroglia [31]. This promotes brain repair by stimulating the proliferation of neural stem cells and the regeneration of damaged tissue. The LIF also has been proven to limit the demyelination of oligodendrocytes in mouse models with autoimmune diseases like encephalomyelitis [34]. Apart from the three main activating pathways that the LIF activates to promote neuroprotection (MAPK, PI3K/Akt, and JAK/STAT), studies have shown that cells treated with the LIF increased their activity of antioxidant enzymes in oligodendrocytes and neurons, decreasing the oxidative damage which leads to cell survival [31]. The leukemia inhibitor factor modulates a variety of CNS responses to inflammatory stimuli. The LIF is actively transported across the BBB, and neuroinflammation is a facilitator for the LIF to enter the brain [34]. Animal models with neuroinflammatory diseases have shown that in response to lipopolysaccharide (LPS), a proinflammatory cytokine, microvessels increased their expression of gp130, suggesting that the CNS increases the permeability of the BBB to increase the transport of the LIF to the site of injury when compared with healthy mice [34].

The neuroprotective effects of the LIF have been explored to understand if it may have the same benefits in the PNS. The discovery of modulating factors on peripheral nerve regeneration, such as nerve growth factor (NGF), set a stepping zone in knowledge about the auto capacity of peripheral nerves to heal [35]. However, the recovery of functionality after a nerve injury can be a tedious process, negatively impacting the quality of life of the affected individual. Taking this into consideration, understanding if the LIF modulates axonal regeneration after a nerve injury can open up a new perspective for future therapies. Schwann cells are the main glial cells in charge of migrating to the site of injury to begin the regeneration and repair of affected neurons [36], but the question remains if the LIF can modulate the action of Schwann cells in axonal myelination and thus speed up the recovering of nerve function [37]. A study on sciatic nerve injury in animal models showed that when the LIF knockout genes were inserted into injured peripheral nerves and the proliferation and migration of Schwann cells increased, this led to enhanced boosted myelinization and debris removal compared with elevated LIF expression in control nerves post-injury, suggesting that the LIF has an inhibitory effect on modulating the actions of glial cells in the peripheral nervous system, having an opposite effect on the neuroregeneration seen in the CNS [37]. The duality of the effect of the LIF among the CNS and PNS is a particularity of this cytokine and its capacity to exhibit different effects on different cell types and cellular statuses.

### 1.5. Experimental Data Indicating LIF’s Potential Role in HIV Neuroprotection

The leukemia inhibitory factor (LIF) has emerged as a multifaceted protective agent, playing pivotal roles in neuroprotection, viral replication, and ocular health. Davis et al. (2019) [38] elucidated that in a rat model of stroke, LIF’s neuroprotective effects hinged on the transcription factor myeloid zinc finger-1 (MZF-1). LIF administration enhanced MZF-1 protein levels, and its neuroprotective attributes were thwarted when MZF-1 was suppressed in vitro. On a different front, Tjernlund et al. (2007) [39] identified LIF’s capacity to inhibit HIV-1 replication by intervening with the Jak/Stat signaling pathway, a conduit HIV-1 harnesses. This interference manifests as diminished HIV-1-mediated Stat 3 phosphorylation, curtailing the virus’s replication. In the realm of ocular health, LIF’s role in neuroprotection becomes evident in its counteraction against acute ocular hypertension (AOH), a precursor to glaucoma. LIF injections in an AOH rat model remarkably ameliorated retinal damage and swelling. This restoration was accompanied by reduced retinal ganglion cell loss and diminished apoptotic markers. The molecular underpinning of this effect was traced back to the augmented activation of STAT3 and mTOR/p70S6K signaling pathways, with the significance of these pathways emphasized when their inhibition reversed LIF’s benefits [38,39].

Adding to LIF’s protective spectrum in the ocular arena, recent studies carried out by Dong et al. (2021) [40] have spotlighted its potential as a neuroprotective agent against oxidative damage in photoreceptor cone cells. An in vivo model with dark-adapted mice exposed to bright light illustrated LIF’s prowess in shielding cone cells from light-induced harm. This protective mantle is tethered to the activation of the Janus tyrosine kinase (JAK)/STAT3 signaling pathway and subsequent modulation of genes related to apoptosis and proliferation. This assertion finds further backing from in vitro experiments using 661 W cells subjected to H_2_O_2_. Where H_2_O_2_ heralded oxidative stress and apoptosis, LIF preemptively curtailed these adverse outcomes. A notable observation was the dwindling of LIF’s protective effects upon the introduction of an STAT3 inhibitor, reiterating the instrumental role of the STAT3 pathway in LIF’s neuroprotective schema. Conclusively, the data amplify LIF’s ability to counter oxidative damage in cone cells via the STAT3 pathway, augmenting its therapeutic versatility across neural, viral, and ocular contexts [40].

In the context of glaucoma, a leading cause of irreversible blindness, neuroprotection remains a vital therapeutic approach. Highlighting the potential of leukemia inhibitory factor (LIF)—an IL-6 cytokine family member known for its role in retinal neuroprotection—a recent study explored its effects on acute ocular hypertension (AOH). This model, established by elevating intraocular pressure in rat eyes, demonstrated the damaging consequences of AOH, including tissue swelling and structural retinal damage. However, post-AOH LIF injections remarkably reversed these adverse outcomes. Notably, LIF treatment significantly curtailed the loss of retinal ganglion cells (RGCs), reduced apoptosis markers, and downregulated proteins linked to cell death. This therapeutic efficacy appeared to stem from the activation of the STAT3 and mTOR/p70S6K signaling pathways. Yet, when inhibitors targeting these pathways were applied, LIF’s protective benefits were nullified. These findings underscore LIF’s potential as a promising agent for neuroprotection in glaucoma management, mediated through specific cellular pathways [41].

## 2. HIV-Associated Neurocognitive Disorders and ART

HIV is involved in the pathogenesis of distinct neurological disorders. While the mechanisms underlying HIV’s ability to invade the CNS remain elusive, it is established that during the initial phases of infection, HIV-infected macrophages circulating in the bloodstream possess the capability to cross the blood–brain barrier. Consequently, macrophages and microglial cells are the primary cellular targets of HIV within the brain, and the neurotoxic effects stem from the release of viral proteins by these cells [42].

HIV can induce several neuropathological processes in the brain, including neuroinflammation, neuronal apoptosis, and synaptic dysfunction. Neuroinflammation develops as a result of an inflammatory cascade initiated by the release of chemokines and cytokines, which can cause harm to neurons and disrupt their physiological functioning. The leukemia inhibitory factor (LIF) is a cytokine released due to neuroinflammation, promoting cell differentiation as a way to protect the central nervous system. As HIV cannot directly infect neurons, it secretes viral proteins, such as Tat (transactivator of transcription) and gp120 (an envelope protein situated in the external membrane of the HIV molecule), to elicit neuronal injury [43]. These viral proteins are taken up by neurons, which leads to axonal damage. Consequently, impaired neurogenesis, neuronal apoptosis, and synaptic dysfunction manifests, thereby establishing a correlation with HIV-associated neurocognitive disorders.

Two explanatory models are proposed regarding how the neurodegeneration that leads to the development of the symptoms of HIV-associated neurocognitive disorders (HANDs) occurs. The direct model posits that neuronal death is caused by a direct interaction between viral proteins and neurons. However, the indirect model states that neuronal death occurs due to an inflammatory reaction carried out by inflammatory cells that act against the HIV proteins released by infected cells. Macrophages and microglial cells activated during the inflammatory reaction are capable of secreting mediators during the process. However, although some of these mediators have neuroprotective characteristics, others can become neurotoxic and can even impede the neuroprotective functionality of astrocytes, which increases the possibility of astrocytic apoptosis [44].

The term HANDs is an umbrella one that covers three subgroups: asymptomatic neurocognitive impairment (ANI), where there are two or more cognitive abnormalities without any functional impairment; mild neurocognitive disorder (MND), which presents cognitive impairment with mild functional impairment; and HIV-associated dementia (HAD), where there is marked cognitive and functional impairment [44,45], as represented by Table 1. To make an appropriate diagnosis, neuropsychological examinations and an evaluation of functional status are used [46]. Researchers are beginning to evaluate certain molecules present in HIV patients as possible biomarkers for HANDs. Since neuronal damage is correlated to the development of HANDs, biomarkers for neuronal injury are studied. For example, the neurofilament light chain (NFL) is a blood biomarker for neurodegenerative diseases. In HIV patients presenting HANDs, NFL levels are higher than in patients without HANDs, and levels continue to increase while the disease progresses. Therefore, NFL concentrations in the cerebral spinal fluid predict the development of HAD [47].

## 3. The Rise of ART

Antiretroviral therapy (ART) was introduced in 1996 as a form to decrease the morbidity and mortality of HIV-infected individuals improving the immune response. Nevertheless, HANDs are still very prominent despite the efforts and improvements in ART. ART divides drugs which have different mechanisms of attack in the HIV cycle and are approved by the Federal and Drug Administration (FDA) into different classes. The classes are divided into NRITs, NNRTIs, PI, fusion inhibitors, CCR5 antagonist, INSTIs, attachment inhibitor, post-attachments inhibitors, capsid inhibitors, and pharmacokinetic enhancers. Table 2 depicts a classification of ARTs by neuroprotective properties.

### 3.1. Non-Neuroprotective ARTs

Nucleoside reverse transcriptase inhibitors (NRTIs) are agents that block the enzyme reverse transcriptase binding competitively, causing premature DNA chain termination. All NRTIs have neurotoxic potencies [49]. They are known to cause mitochondrial toxicity, which is known to target the main organs involved in drug metabolism or that rely on oxidative phosphorylation such as the brain [50]. This may cause neurotoxicity, brain morphology, neurological anomalies, cognitive impairment, and episodes of seizures in children, especially those children exposed to Azidothymidine (AZT) [51]. AZT is used to reduce vertical transmission of HIV. AZT does not cross the blood–brain barrier (BBB) but it accumulates in the cerebrospinal fluid (CSF), reducing the population expansion potential and neurogenesis of neuronal/stem progenitors [52]. Emtricitabine (FTC) and Tenofovir (TDF) are the most used NRTIs in the US and other developed countries [53]. However, studies have found that FTC and TDF cause damage in the nervous system, such as beading, simplification of the dendritic processes, and neuronal shrinkage [54]. Non-nucleoside reverse transcriptase (NNRTI) binds to the reverse transcriptase in a noncompetitive hydrophobic site not requiring phosphorylation. Efavirenz (EFZ) is the most used agent of this class [53]; yet, it has been found that EFZ alters mitochondrial respiration in neurons and glial cells, decreasing the mitochondrial potential membrane and causing an increased generation of mitochondrial reactive oxygen species exacerbated in neuroinflammatory conditions, causing effects in the central nervous system (CNS) [55]. The CNS effects include, but are not limited to, vivid dreams, delusions, sleep disturbance, dizziness, headaches, increased suicidality, psychosis-like behavior, and mania. There was another study on Rilpivirine (RPV), another NNRTI, that found that it does not affect mitochondrial function or compromise cell viability and survival in hepatic cells and neurons [56]. Theoretically, this does not affect the CNS, but it does not provide neuroprotection either. Protease inhibitors (PIs) competitively inhibit the proteolytic cleavage of the gag/pol polyproteins in HIV-infected cells [50]. The delivery of protease inhibitors to the brain is limited due to the multidrug-resistance proteins expressed on endothelial cells [57]. Therefore, even if the viral load is well controlled, once the virus is in the brain it can cause neural dysfunction. Also, PI does not impact the production of early gene products such as Tat, Nef, and Rev. If Tat is released, it can induce effects into the brain, producing dendritic loss [58].

### 3.2. Neuroprotective ARTs

Fusion inhibitors block viral uptake by binding to the gp41 envelope structure. Studies have found that enfuvirtide (T20), a biomimetic peptide, provides potent protection against neuronal cell destruction by a variety of HIV strains [59]. This is only achieved if T20 is combined with another ART that has adequate CNS protection. If not, T20 on its own enables viral replication in CSF, leading to a subsequent selection of enfuvirtide drug resistance [60]. CCR5 antagonist selectively and reversibly blocks entry into the CD4 T-cells by preventing interaction between CD4 cells and the gp120 subunit of the viral envelope glycoprotein [50]. Maraviroc (MVC), a CCR5 antagonist, is being targeted for the treatment of neuroinflammatory disorders [61]. HANDs may be caused by circulating bone-marrow-derived monocytes infected by HIV that pass through the BBB, triggering neuroinflammation and leading to neuronal degeneration. However, it has been found that MVC favorably alters monocyte activation, improving neuropsychological improvement [62]. Integrase strand transfer inhibitors (INSTIs) inhibit the integration of viral DNA into the chromosomal host DNA. Raltegravir (RAL) is an INSTI agent with low toxicity [61] that is being studied as a potential neuroprotective agent against HIV. A study found that RAL is anti-inflammatory when administered in healthy microglia. However, when RAL interacts with HIV-infected microglia, it is anti-inflammatory. This may be explained by the presence of the HIV-replication complex as it is bound and not interacting with off-target proteins [63]. This means that it can prevent HIV-infected macrophages from crossing the blood–brain barrier and causing neuronal damage. Other studies have found that RAL does not induce any alteration in mitochondrial function, making it a safer neurological profile [56]. Pharmacokinetic enhancers are used in HIV treatment to increase the effectiveness of an HIV medicine included in an HIV treatment regimen. [64] Cobicistat (COBI) lacks antiretroviral (ARV) activity; however, it has been shown in vitro that COBI inhibits p-glycoprotein [65]. Patients with HIV-associated dementia (HAD) brains show that P-glycoprotein is overexpressed in the blood–brain barrier, causing a low permeability to successful antiretroviral treatments like protease inhibitors [66].

### 3.3. ART in Phase III

Attachment inhibitors interfere with the gp120 protein on the outer surface of HIV [64]. Fostemsavir (FTR), in a Phase III study, is showing promise; the final results should be posted in 2024 [67]. In the BRIGHTE study, the side effects of FTR were studied in patients in the first 24 weeks. Nine patients experienced serious side effects. One of those side effects was immune reconstitution inflammatory cases, which presented neurotoxoplasmosis, central nervous system lesion, and atypical mycobacterial infection [68]. Nevertheless, more studies need to be conducted to understand more of its side effects. Post-attachments inhibitors block CD4 receptors on the surface of certain immune cells that HIV needs to enter the cells [64]. Ibalizumab-uiyk (Trogarzo) is currently in a Phase III study. In clinical trials, it has been found that IBA is a great ART therapy for patients with multidrug-resistant HIV infection. However, additional studies have to be conducted to obtain long-term data and understand its efficacy [69] and the role it plays in the neuroprotection of neurotoxicity. Capsid inhibitors interfere with the HIV capsid or shell, preventing future replication [64]. Lenacapavir (GS-HIV) is within a Phase III study; it is being evaluated as a possible Prep. However, the results of the prevention trials and their side effects are still not available [70].

## 4. Introducing cART in the Treatment of HANDs

HANDs are an impairment in the cognitive, behavioral, and motor functions of an HIV patient. Even though there have been many breakthroughs through the help of antiretroviral therapy, many patients living with HIV still experience the severe symptoms of HANDs [71]. The lack of an effective treatment is due to constant immune system activation, which in turn increases the risk. Another limitation is the incomplete protection provided by ART to the CNS, leading to the development of HANDs [72]. Antiretroviral therapy (ART) is intended to lower and maintain low viral levels, improve immune system function by increasing CD4+ T-cell counts, and reduce HIV-associated morbidity [73]. Essentially, even patients undergoing ART treatment are at risk for HANDs [72]. However, it has been found that combination antiretroviral therapy (cART) is an effective intervention for reducing the prevalence of patients with HIV-associated neurocognitive disorders [61].

## 5. Combination Antiretroviral Therapy (cART)

Over the past years of research, ART has evolved to cART. They combine different ARTs to provide a better treatment plan for each patient’s needs [74]. Combination antiretroviral therapy is the use of a combination of multiple pharmaceutical agents, outlined in Table 2, to attack the HIV virus by various life cycle checkpoints, thus decreasing the damage being caused while simultaneously decelerating its development. They are able to achieve this with the help of reverse transcriptase inhibitors as these are able to bind and block HIV reverse transcriptase, thereby stopping its replication. By effectively controlling HIV replication, cART helps to preserve immune function, including in the CNS. This can prevent the development and progression of HANDs.

It has been shown that the introduction of cART has led to a decline in the overall prevalence of severe forms of HANDs. Before the advent of effective antiretroviral therapy, HANDs were more common and often progressed to more severe stages. However, with the widespread use of cART, the incidence and severity of HANDs have decreased.

## 6. Viral Suppression and the Risk of Developing HANDs

**Figure 1:**
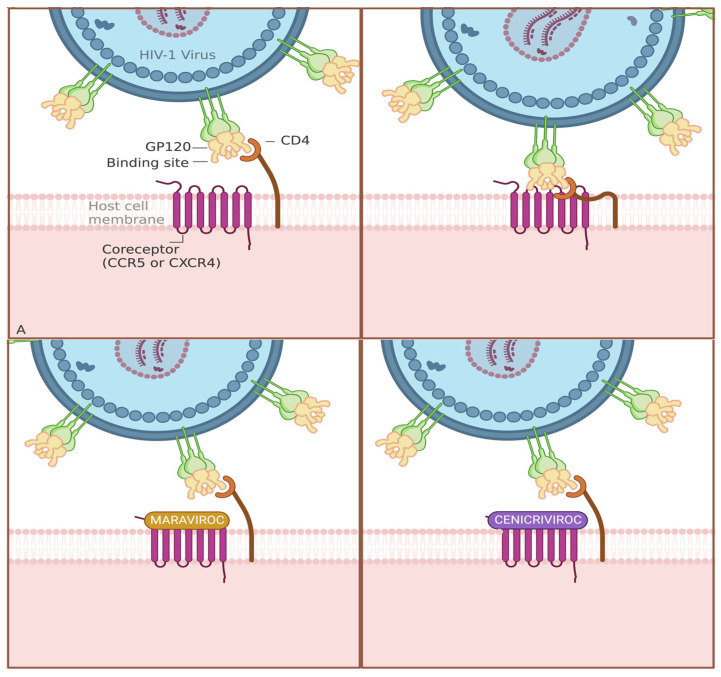

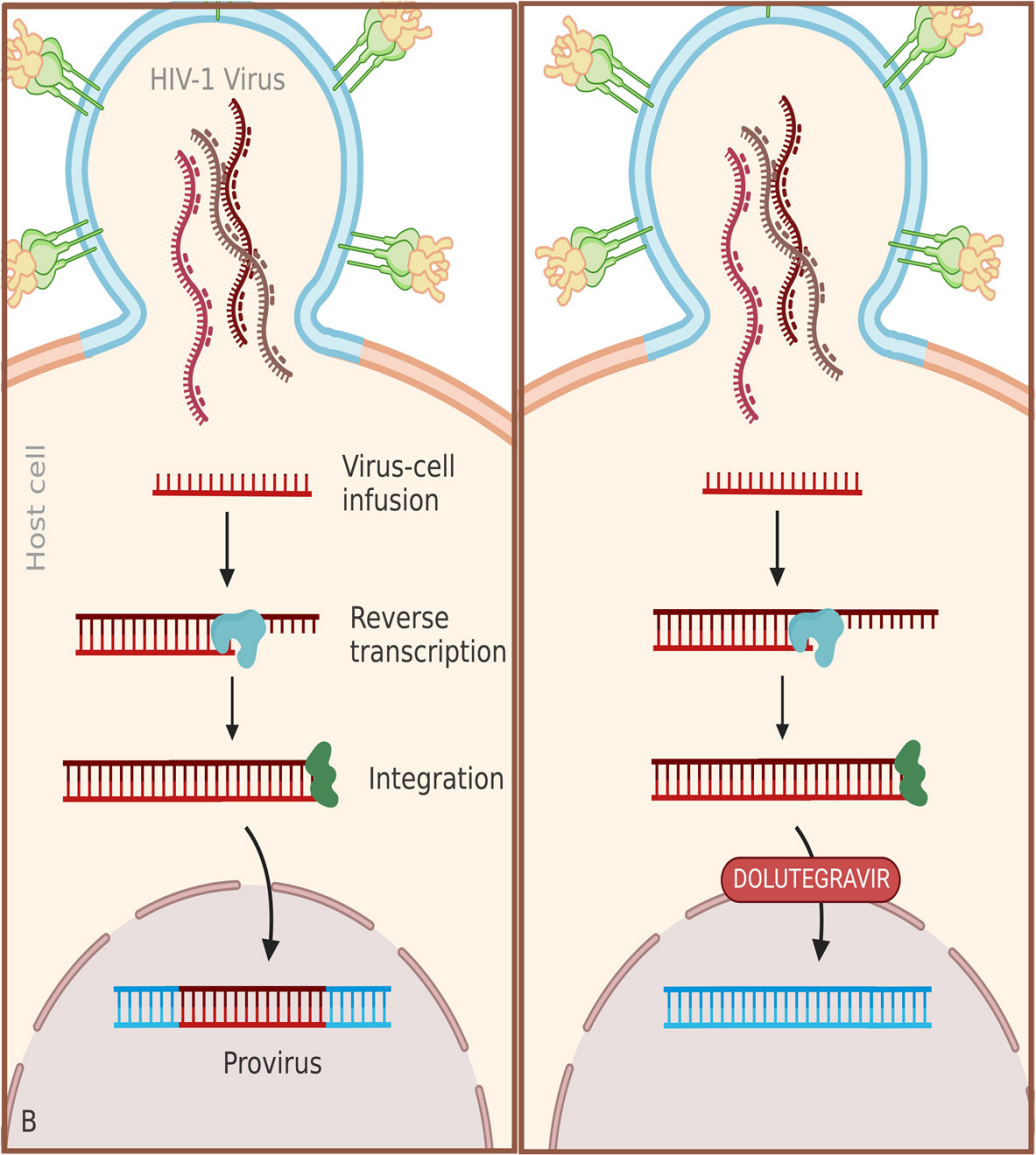
Mechanism of action for common cARTs that provide neuroprotection. (**A**) MVC and CVC work as an antagonist by blocking the CCR5 or CCR2 co-receptors on immune cells and preventing HIV from entering and infecting them. By blocking these co-receptors, there is a potential decrease in inflammatory responses. (**B**) A virus-cell infusion scenario. Dolutegravir is an integrase inhibitor that targets the integrase enzyme essential for viral replication. By blocking the integrase active site, it prevents the integration of viral DNA into the host cell’s genome.

Viral suppression, achieved through cART, is crucial in reducing the risk of developing HANDs. Higher levels of viral replication and uncontrolled HIV infection have been associated with an increased risk of neurocognitive impairment. Blockers such as protease inhibitors, entry inhibitors, and maturation inhibitors, as well as reverse transcriptase inhibitors and integrase inhibitors, are all possible ways cART has been shown to help maintain a lower HIV viral load in the bloodstream and CNS. Maraviroc (MVC) and Cenicriviroc (CVC) are antiretroviral therapeutics and fusion inhibitors whose dosage intensification has led to a decrease in monocyte HIV DNA levels and immune activation in infected cells. This effect was more significant in subjects with cognitive impairment, suggesting that MVC and CVC may have a positive impact on cognitive function in HANDs [62,75]. MVC’s and CVC’s mechanism of action is represented in Figure 1A. Dolutegravir is an integrase inhibitor used in HIV treatment that may offer potential neuroprotection by targeting the integrase enzyme essential for viral replication, potentially mitigating the neurotoxic effects associated with HIV [76,77]. The neuroprotective mechanism, as represented in Figure 1B, could involve the prevention of direct neurotoxicity from viral replication and reduction in oxidative stress and neuronal damage, as well as diminishing cerebral degenerative changes [78]. As mentioned by suppressing viral replication, cART helps maintain lower HIV viral loads in the blood and CNS. This reduces the inflammatory response and neurotoxicity caused by HIV, which are contributing factors to the development of HANDs. Therefore, individuals who achieve and maintain viral suppression with cART have a lower risk of developing HANDs compared to those with uncontrolled HIV replication. Interestingly, it is worth noting that some attempts to increase cART’s presence to decrease viral toxicity have been shown to increase ART neurotoxicity and neuropsychiatric effects.

## 7. Conclusions

As mentioned previously, HANDs hinder the quality of life of HIV patients. The severity of HANDs is determined by the number of neurological processes that are affected, through neuropsychological screening (Table 1). These cognitive impairments result from constant neuroinflammation that disrupts the physiological baseline. Degenerative neurological processes lead to gradual brain atrophy in the subcortical regions, including deep frontal white matter and basal ganglia [79]. On account of the neurodegenerative process, it can be expected that HIV patients suffer alterations in basic cognitive skills such as memory, attention, learning, concentration, and motor skills [48]. The progression of HANDs has been measured using an MRI to quantify brain atrophy volume. A study reported that 32% of their subjects were diagnosed with ANI following neurocognitive tests as well as an MRI. The patients participated poorly in the neurocognitive tests, and the MRI showed diminished brain atrophy as a whole [80]. This demonstrates that neurocognitive impairments are, in part, due to structures losing functionality as they atrophy.

The chronic treatment of HANDs continues to be an ongoing threat to the affected populations. ARTs have been proven to successfully diminish HIV viral loads to near undetectable viral levels and improve CD4+ T cell counts. However, reducing the prevalence of HANDs has proven to be strenuous. Currently, 50% of patients treated with cART develop mild neurocognitive symptoms. Although it has halved the prevalence of HAD, it has resulted in a higher prevalence of mild neurocognitive symptoms [81]. Nonetheless, cART is still an invaluable resource in the management of HIV and HANDs. It is advantageous to use a combination of ART to further target various points of the lifecycle to enhance suppression and minimize resistance [82]. The addition of the LIF could serve to preserve neurocognitive functions in patients suffering from HANDs. As we examined previously, the LIF is a modulating cytokine with many functions that could prove to be beneficial by stimulating neurogenesis, resulting in a halt of neurocognitive decline.

A novel approach is needed to accurately detect HANDs in the compromised HIV community. Imaging technologies serve to detect the abnormal deterioration of brain mass in suspected HAND patients. However, when combined with an aging population, such comorbidities could interfere with a reliable diagnosis [83]. More research has to be conducted to find an accurate biomarker with which to effectively detect HANDs and to provide tailormade cARTs. Studies have garnered attention towards biomarkers that indicate the presence of neuronal injury, specifically CSF, such as NFL chains, which have been reported to be expressed at high levels even during ongoing cART treatments [84]. Furthermore, in a recent study, plasma miRNA was used to predict and detect HANDs in cohorts of MND and HAD [85]. If explored thoroughly, it could provide a reliable screening method for the detection of HANDs. Early detection could significantly reduce the severity of neurological decline, thereby improving the quality of life for these individuals.

## Figures and Tables

**Table 1:** Criteria for HIV-associated neurocognitive disorders based on the Frascati criteria.

	Neurocognitive Condition	Functional Status
Asymptomatic neurocognitive impairment (ANI)	1 SD below mean in cognitive domain assessments	No interference with lifestyle
Mild neurocognitive impairment (MND)	1 SD below mean in cognitive domain assessments	Mild interference with lifestyle
HIV-associated dementia (HAD)	2 SD below mean in cognitive domain assessments	Notable interference with lifestyle

Neurocognitive condition is determined by measuring 2 ability domains along with their performance to be compared with mean age–education. Performance scores noting at least a standard deviation (SD) of 1.0 are documented. Neurophysiological assessment includes verbal/language; attention/working memory; abstraction/executive; memory, learning, recall; speed of information processing; sensory–perceptual; and motor skills. The table is adapted from Antinori et al., 2007 [48].

**Table 2:** Compact list of ARTs displaying neuroprotection.

Class of ART	Neuroprotection	No Neuroprotection	Effects
NRTIs	-	✓	Mitochondrial toxicity, neurotoxicity, brain morphology, neurological anomalies, cognitive impairments, cognitive impairment, seizures in children, reduces the population of potential neurogenesis, simplification of the dendritic process, and neuronal shrinkage.
NNRTIs	-	✓	Alters mitochondrial respiration in neurons and glial cells, vivid dreams, delusions, sleep, disturbance, dizziness, headaches, increased suicidality, psychosis-like behavior, and mania.
PI	-	✓	Dendritic loss due to released Tat, multidrug-resistance in the brain.
Fusion inhibitor	✓	-	Protection against neuronal destruction.
CCR5 antagonist	✓	-	Protection against infected bone-marrow-derived monocyte that crosses the BBB.
INSTI	✓	-	Low toxicity with neuroprotective agents against microglia infected with HIV.
Attachment inhibitor	-	-	Unknown
Post-attachment inhibitor	-	-	Unknown
Capsid inhibitor	-	-	Unknown
Pharmacokinetic enhancers	✓	-	Inhibits p-glycoprotein in vitro.

The list exemplifies ARTs mentioned in the text body. ARTs are classified by benefit of interest, neuroprotection, or lack thereof. A short summary is also provided regarding mechanisms of action. “✓” indicates possession of classification.

## Data Availability

Not applicable.
